# Association Between Circulating Linoleic Acid and Risk of Ischemic Stroke

**DOI:** 10.3389/fgene.2020.582623

**Published:** 2021-01-08

**Authors:** Ding Ye, Huijun Huang, David J. H. Wu, Wanting Zhang, Feixiang Zhou, Yu Qian, Jusheng Zheng, Yingying Mao

**Affiliations:** ^1^School of Public Health, Zhejiang Chinese Medical University, Hangzhou, China; ^2^School of Medicine, University of Minnesota, Minneapolis, MN, United States; ^3^School of Life Sciences, Westlake University, Hangzhou, China

**Keywords:** Genetic variation, ischemic stroke, linoleic acid, Mendelian randomization, single nucleotide polymorphism

## Abstract

**Background:**

Observational studies have shown an inverse association between circulating linoleic acid (LA) and risk of ischemic stroke (IS).

**Objective:**

The aim of this study was to explore whether genetic variants predicting levels of circulating LA are associated with IS and its subtypes using a two-sample Mendelian randomization (MR) analysis.

**Methods:**

LA-related single-nucleotide polymorphisms (SNPs) were selected from a genome-wide association study of 8,631 participants, and summary statistics of IS and IS subtypes were obtained from the MEGASTROKE consortium. MR analysis was performed using the inverse-variance weighted (IVW) method complemented with other approaches, including weighted-median, weighted-mode, MR Pleiotropy RESidual Sum and Outlier test and MR-Egger regression, to test for the robustness of the association. Moreover, we conducted bidirectional MR analysis to assess the impact of IS-associated SNPs on circulating LA levels. Odds ratios (ORs) with 95% confidence intervals (95% CIs) were calculated.

**Results:**

We found that genetically predicted circulating LA levels were inversely associated with the risk of IS by the IVW method (OR = 0.98, 95% CI: 0.97–0.99, and *P* = 0.003). Subgroup analyses showed a statistically significant association between LA and risk of large artery stroke (LAS; OR = 0.95, 95% CI: 0.92–0.98, and *P* = 0.004), but not for other IS subtypes. The results were stable in sensitivity analyses, and no evidence of reverse association between LA and risk of IS, or LAS was observed.

**Conclusion:**

Our study supports a potential inverse association of genetically predicted circulating LA levels with risk of IS, particularly LAS.

## Introduction

Stroke is one of the leading causes of mortality and disability worldwide (Hankey, 2017). Ischemic stroke (IS) is the most common type of stroke, and its global burden is projected to grow (Roth et al., 2017). Modifiable lifestyle factors have been widely explored in IS prevention, including diet (Larsson, 2017). Linoleic acid (LA) is one of the primary dietary omega-6 polyunsaturated fatty acids essential for various physiological functions, such as cell signaling and lipid metabolism (Whelan and Fritsche, 2013). To be noted, circulating LA is considered as a valid biomarker for dietary LA intake (Hodson et al., 2008).

Observational studies have reported an inverse relationship between circulating LA level and IS. Recently, an individual-level pooled analysis of 21 cohort studies from 13 countries demonstrated that higher circulating LA level was associated with a lower risk of incident IS (Marklund et al., 2019). However, because of the presence of potential confounding and reverse causality in observational studies, it is difficult to distinguish whether the observed association between circulating LA and IS risk is causal or not. Unfortunately, there is no randomized controlled trial assessing the effect of LA on IS so far. Moreover, the associations of LA with IS subtypes have not been investigated in depth.

Mendelian randomization (MR) is a genetic epidemiological approach that can overcome the bias of potential confounding and reverse causation by using genetic variants, usually single nucleotide polymorphisms (SNPs), as instrumental variables (IVs) to infer causality (Burgess et al., 2017b). As genetic variants cannot be modified by disease status, reverse causation could be avoided in MR analyses. Moreover, as genetic variants are presumed to be randomly distributed in the general population according to Mendel’s laws of inheritance, independence confounders, MR analyses can minimize the risk confounding bias (Burgess et al., 2015a,b).

Therefore, in the current study, we performed a two-sample MR analysis to assess whether genetically predicted circulating LA levels were associated with risk of IS and its subtypes.

## Materials and Methods

### Data Source

**FIGURE 1:**
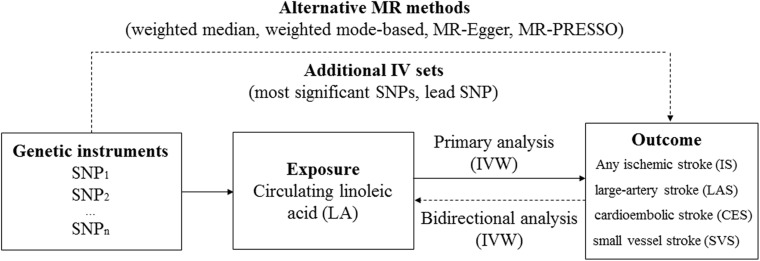
An overview of the Mendelian randomization (MR) study design. Abbreviation: IVW, inverse-variance weighted; PRESSO, Pleiotropy RESidual Sum and Outlier.

An overview of the study design is shown in Figure 1. The genotype data for IS was obtained from the MEGASTROKE consortium (http://megastroke.org/) (Malik et al., 2018). Details of this study have been described elsewhere. Briefly, the summary statistics were derived from 438,847 individuals of European ancestry (34,217 cases with IS and 404,630 controls). The study identified 32 loci associated with risk of IS, which were used as genetic instruments for subsequent bidirectional MR. According to the Trial of Org 10172 in Acute Stroke Treatment criteria (Adams et al., 1993), cases with IS were further classified into the cases with large-artery stroke (LAS; *n* = 4,373), cardioembolic stroke (CES; *n* = 7,193), and small vessel stroke (SVS; *n* = 5,386).

### Selection of Instrumental Variables

A recent genome-wide association study (GWAS) including 8,631 participants of European ancestry (Mean age: 60 years old; Male: 45%) reported 173 SNPs associated with circulating levels of LA at genome-wide significance threshold (*P* < 5 × 10^–8^; Guan et al., 2014). We checked the pairwise linkage disequilibrium (LD) in terms of *r*^2^ using the LD calculator (https://analysistools.nci.nih.gov/LDlink/) based on 1000 GENOMES: phase_3: CEU population, and 11 independent SNPs (*r*^2^ < 0.1 and D’ < 0.8) were selected and used as IVs in the primary analysis (Supplementary Table 1). One variant, rs1800009, was not available from IS summary statistics and was replaced by its proxy, rs3758977 (*r*^2^ = 0.97). Moreover, we checked whether the selected SNPs were associated with other traits using the GWAS Catalog (https://www.ebi.ac.uk/gwas/, accessed on Nov 17, 2020), and performed the sensitivity analysis by excluding SNPs related to other phenotypes.

Furthermore, we used additional SNP sets as IVs (Supplementary Table 2) and assessed the association between circulating LA levels and risk of IS and its subtypes. Specifically, we used three most significant independent SNPs associated with circulating levels of LA as IVs (Guan et al., 2014). Subsequently, we repeated the analyses only using the lead SNP (rs174547, *P* value with LA: 4.98 × 10^–274^) to further rule out the possibility of bias due to inclusion of invalid SNPs.

### Statistical Analysis

Mendelian randomization analysis was performed using the MendelianRandomization package in R software (version 3.5.2). The odds ratios (ORs) with 95% confidence intervals (95% CIs) of IS were scaled to the change of LA in percentage of total fatty acids.

The primary analyses were conducted using the inverse-variance weighted (IVW) method under the random-effects model (hereafter referred to as standard MR analysis), which combined effect estimates for each SNP and provided estimates of circulating LA levels on IS and its subtypes to calculate the Wald estimates when all included genetic variants satisfied the valid IV assumptions (Burgess et al., 2017a). IVW uses an approach analogous to meta-analysis to combine these Wald estimates (Palmer et al., 2011). The standard error of the Wald estimate is calculated by the delta method (Burgess et al., 2013).

In sensitivity analyses, we first used weighted median and weighted mode-based methods. The weighted-median method provides consistent estimates of the causal effect if more than half of weight is derived from valid IVs. However, weighted mode-based method is consistent even if the majority of instruments are invalid. We also used MR-Egger regression to identify and adjust for potential pleiotropy effects (Hartwig et al., 2017), which is disposed to regression dilution bias. The average horizontal pleiotropic effect across all genetic variants can be interpreted as an estimate. In addition, the MR Pleiotropy RESidual Sum and Outlier (MR-PRESSO) tests were used to identify potential outliers (Verbanck et al., 2018). Bidirectional MR analysis was also performed to assess the impact of IS-associated SNPs on circulating LA levels.

The effect detected by the sample size to provide 80% statistical power at an alpha level of 5% was computed using the online mRnd power tool at https://shiny.cnsgenomics.com/mRnd/. With the least variance explained by the lead SNP (7.6% variance, Guan et al., 2014) and the sample size of MEGASTROKE, there was >80% power to detect associations with IS for circulating LA at a smallest effect size (OR) of 0.94. Power were lower for IS subtypes, and the corresponding ORs were 0.84, 0.88, and 0.86 for LAS, CES, and SVS, respectively.

## Results

### MR Estimates by Eleven Instrumental SNPs

Associations of the eleven LA-associated SNPs with risk of IS and its subtypes are shown in Supplementary Table 3, and the scatter plot is presented in Supplementary Figure 1. In the primary MR analyses, we found that genetically predicted circulating LA levels were inversely associated with risk of IS (OR: 0.98, 95% CI: 0.97–0.99, and *P* = 0.003). In the subgroup analysis, genetically predicted circulating LA was associated with a reduced risk of LAS (OR: 0.95, 95% CI: 0.92–0.98, and *P* = 0.004; Table 1). However, no statistically significant association was observed for circulating LA levels with risk of CES (OR: 0.98, 95% CI: 0.96–1.00, and *P* = 0.090) or SVS (OR: 1.01, 95% CI: 0.98–1.04, and *P* = 0.340).

**TABLE 1:** Effect estimates of the associations between genetically predicted linoleic acid (LA) and ischemic stroke and its subtypes.

**Outcomes and methods**	**Number of SNPs**	**OR (95% CI)**	***P*-value for association**
**IS**			
IVW	11	0.98 (0.97–0.99)	0.003
MR-Egger	11	/	0.797^a^
Weighted median	11	0.98 (0.97–1.00)	0.015
Weighted mode-based	11	0.99 (0.97–1.00)	0.030
MR-PRESSO	11	/	0.838^b^
**LAS**			
IVW	11	0.95 (0.92–0.98)	0.004
MR-Egger	11	/	0.060^a^
Weighted median	11	0.94 (0.90–0.97)	0.001
Weighted mode-based	11	0.94 (0.92–0.95)	0.002
MR-PRESSO	11	/	0.100^b^
**CES**			
IVW	11	0.98 (0.96–1.00)	0.090
MR-Egger	11	/	0.601^a^
Weighted median	11	0.98 (0.96–1.01)	0.180
Weighted mode-based	11	0.98 (0.96–1.01)	0.226
MR-PRESSO	11	/	0.571^b^
**SVS**			
IVW	11	1.01 (0.98–1.04)	0.340
MR-Egger	11	/	0.864^a^
Weighted median	11	1.02 (0.99–1.05)	0.201
Weighted mode-based	11	1.02 (0.99–1.05)	0.320
MR-PRESSO	11	/	0.397^b^

*CES, cardioembolic stroke; CI, confidence interval; IS, ischemic stroke; IVW, inverse-variance weighted; LA, linoleic acid; LAS, large artery stroke; OR, odds ratio; PRESSO, Pleiotropy RESidual Sum and Outlier; and SVS, small vessel stroke.*

*^a^*P* value for the intercept from MR-Egger regression analysis.*

*^b^*P* value of MR-PRESSO global test.*

In the sensitivity analysis using alternative MR methods, similar effect estimates were observed using the weighted-median and weighted mode-based methods (Table 1). MR-Egger regression did not indicate the presence of directional pleiotropy for IS and its subtypes (All *P* > 0.05). Moreover, no outlier SNPs were detected using the MR-PRESSO test (All *P* > 0.05).

### MR Estimates by Additional IV Sets

In search of the GWAS Catalog for the eleven instrumental SNPs, three SNPs were found to be associated with potential secondary traits (Supplementary Table 4). After exclusion of these SNPs, the association between circulating LA levels and risk of IS remained statistically significant (OR = 0.97, 95% CI: 0.95–1.00, *P* = 0.024, by IVW method).

**FIGURE 2:**
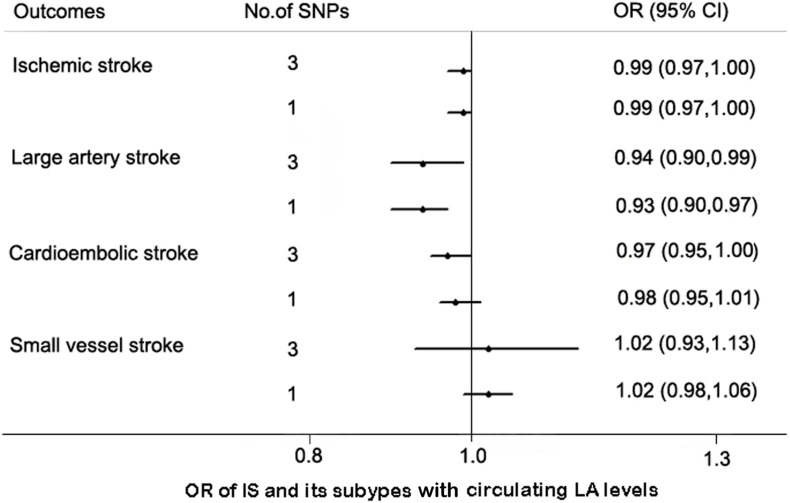
MR estimates between the association of linoleic acid (LA) and ischemic stroke (IS) with its subtypes by additional IV sets. Abbreviation: CI, confidence interval; OR, odds ratio.

We used additional sets of SNPs as IVs to assess the robustness of our findings. The associations of the SNPs with IS and its subtypes are shown in Supplementary Table 5. As expected, the effect estimates of circulating LA levels with IS and LAS remained consistent although the 95% CIs were wider by the IVW method. Similarly, circulating LA levels were not associated with CES or SVS (Figure 2).

### Bidirectional MR

We also performed bidirectional MR analysis to assess the effect of IS and its subtypes on circulating LA levels. Genetically predicted IS and its subtypes were not associated with circulating LA levels using the IVW method (for IS, OR: 1.02, 95% CI: 0.91–1.13, and *P* = 0.78; for LAS: OR: 1.02, 95% CI: 0.95–1.10, and *P* = 0.52; for CES, OR: 1.02, 95% CI: 0.97–1.08, and *P* = 0.46; and for SVS, OR: 0.99, 95% CI: 0.90–1.08, and *P* = 0.76, respectively; Supplementary Table 6).

## Discussion

The current study provided evidence that genetically predicted higher circulating levels of LA was associated with a 1.7% lower risk of IS per percentage in total fatty acid increase in LA. Likewise, a previous meta-analysis of prospective studies found that circulating LA was associated with a 12% decreased risk of IS per interquintile range (Marklund et al., 2019). However, the biological mechanism behind the link of circulating LA levels with IS risk is still unclear. It is possible that high intake of LA may reduce the risk of IS through lowering blood lipids and blood pressure (Mensink et al., 2003; Miura et al., 2008; Yaghi and Elkind, 2015; Xie et al., 2016). In addition, LA may reduce platelet aggregation and enhance deformability of erythrocyte cells (Tsai et al., 1994; Bazan-Salinas et al., 2016), both of which may reduce the risk of IS through improving circulation in small blood vessels.

Only a limited number of observational studies have assessed the relationship between LA and different pathophysiological IS subtypes. One case-control study found that circulating LA level was lower in patients with SVS and LAS than that in controls (Park et al., 2009). Another Danish cohort study demonstrated a dose-dependent inverse association between adipose tissue content of LA and the incidence rate of LAS (Veno et al., 2017). In the current MR analysis, we found circulating LA level was negatively associated with risk of LAS, while no statistically significant association was found between circulating LA and risk of CES and SVS. As different IS subtypes may have different risk factor profiles and pathophysiological mechanisms (Traylor et al., 2012), the association between LA and risk of IS might be subtype specific. A possible explanation might be the different impact of cholesterol on the large precerebral arteries and the small cerebral arteries (Tirschwell et al., 2004; Lv et al., 2016). With the aim to further characterize the interplay among LA, cholesterol and the risk of IS with its subtypes, we also perform MR analysis to explore these intermediary associations. We found that low density lipoprotein (LDL) cholesterol was significantly with the risk of IS and LAS, rather than CES and SVS (Supplementary Table 7), which was consistent with the results of a previous MR study (Hindy et al., 2018). Genetically predicted higher circulating LA was associated with lower LDL cholesterol (OR: 0.98, 95% CI: 0.97–1.00, and *P* = 0.005) by using the MAGNETIC NMR-GWAS summary statistics (Kettunen et al., 2016). Moreover, adjustment for LDL cholesterol attenuated the association between LA and the risk of IS with its subtypes (Supplementary Table 8), which suggested that the effect of LA on the risk of IS may be mediated by LDL cholesterol.

One of the major strengths of the present study is that we performed a series of sensitivity analysis to eliminate the influence of potential pleiotropy. We selected eleven SNPs achieving the genome-wide significant threshold as IVs from a large GWAS study of LA, and we also used additional IV sets to further evaluate the potential causal association circulating LA and IS risk. The three SNPs (rs10740118, rs174547, and rs16966952) independently explained the largest proportions of the overall variance in LA ranging from 8.3% to 21.3% across 5 cohorts (Guan et al., 2014). Therefore, our study minimized the risk of “weak instrument bias” (Zheng et al., 2017). Moreover, we conducted bidirectional MR analysis to further rule out the possibility of reverse causation, which provided additional confidence in the association between LA and risk of IS.

However, some limitations should be considered. First, MR analysis are susceptible to bias from pleiotropy. To eliminate the influence of potential pleiotropy, we performed a series of sensitivity analyses. We did not detect the evidence of pleiotropy for the causal association of circulating LA levels with risk of IS or LAS in sensitivity analyses. However, the associations between LA-related SNPs and other potential confounders, such as insulin sensitivity measurement and chronic kidney disease were difficult to assess, because the publicly available GWAS summary statistics are limited. We cannot avoid unknown potential pleiotropy given our current limited understanding in the etiology of the diseases. Second, as we only included participants of European ancestry, our results may not apply to other races, and further studies with more diverse populations are warranted. Nevertheless, since the MR estimates could be confounded by population stratification (Ebrahim and Davey Smith, 2008), using genetic studies for both exposure and outcome among European ancestry could minimize such bias. Third, the standard MR method assumes a linear association between the exposure and outcome. However, we could not detect if the association between circulating LA levels and IS was non-linear and whether there was a threshold effect. Finally, due to the insufficient statistical power for IS subtypes, we cannot rule out the potential causal relationship of circulating LA with risk of CES and SVS.

In summary, the present results found that genetically predicted circulating LA levels were associated with a reduced risk of IS and LAS, suggesting that moderate intake of LA may serve as a possible prophylactic strategy for IS, particularly LAS. Yet, the reported association from MR approach in the present study needs further confirmation by randomized controlled trials in future.

## Data Availability Statement

The datasets presented in this study can be found in online repositories. The names of the repository/repositories and accession number(s) can be found in the article/Supplementary Material.

## Ethics Statement

No human studies are presented in this article.

## Author Contributions

YM, JZ, and DY contributed to the conception and design of the study. HH, WZ, FZ, DW, and YQ contributed to the analysis of data, preparing the figures, and drafting the manuscript. DY, HH, DW, and YM made ciritical revisions of the manuscript. All authors approved the final manuscript.

## Conflict of Interest

The authors declare that the research was conducted in the absence of any commercial or financial relationships that could be construed as a potential conflict of interest.
