# Supplementary material for: Association Between Circulating Linoleic Acid and Risk of Ischemic Stroke
Source: Front Genet. 2021 Jan 8;11:582623. doi: 10.3389/fgene.2020.582623 (PMC7874191; doi:10.3389/fgene.2020.582623)
Supplement: Supplementary file 1 [file Data_Sheet_1.docx]

| **Supplementary Table 1** Basic information of instrumental SNPs in the MR study | | | | |
| --- | --- | --- | --- | --- |
| SNP | Position | Chr | Allele frequency | EA* |
| rs4454603 | 64682756 | 10 | 0.491 | t |
| rs10897169 | 61153899 | 11 | 0.319 | a |
| rs13966 | 61421568 | 11 | 0.475 | t |
| rs174541 | 61322484 | 11 | 0.655 | t |
| rs1800009 | 61486810 | 11 | 0.644 | t |
| rs198475 | 61282647 | 11 | 0.251 | t |
| rs2521568 | 61457509 | 11 | 0.074 | c |
| rs2521572 | 61468051 | 11 | 0.054 | t |
| rs4963466 | 61658702 | 11 | 0.718 | c |
| rs760306 | 61480868 | 11 | 0.243 | t |
| rs16966952 | 15043444 | 16 | 0.309 | a |
| *EA, effect allele from Guan’s GWAS. | | | | |

| \| **Supplementary Table 2** Basic information of the SNPs in the additional IV sets \| \| \| \| \| \| \| \| \| \| --- \| --- \| --- \| --- \| --- \| --- \| --- \| --- \| --- \| \| SNP \| Position \| Gene \| Chr \| Allele frequency \| EA* \|  \| \| \| rs10740118 \| 64771213 \| JMJD1C \| 10 \| c \| 0.440 \|  \| \| \| rs174547 \| 61327359 \| FADS1 \| 11 \| t \| 0.677 \|  \| \| \| rs16966952 \| 15043444 \| NTAN1 \| 16 \| a \| 0.309 \|  \| \| \| *EA, effect allele from Guan’s GWAS. \| \| \| \| \| \| \| |
| --- | --- | --- | --- | --- | --- | --- | --- | --- | --- | --- | --- | --- | --- | --- | --- | --- | --- | --- | --- | --- | --- | --- | --- | --- | --- | --- | --- | --- | --- | --- | --- | --- | --- | --- | --- | --- | --- | --- | --- | --- | --- | --- | --- | --- | --- | --- | --- | --- |

| **Supplementary Table 3** Associations of instrumental SNPs with LA, and IS with its subtypes | | | | | | | | | | | | | | | | | | | | | |  |
| --- | --- | --- | --- | --- | --- | --- | --- | --- | --- | --- | --- | --- | --- | --- | --- | --- | --- | --- | --- | --- | --- | --- |
| SNP | Association with LA | | |  |  | Association with IS | |  | Association with LAS | |  | Association with CES | | | |  | | Association with SVS | | | |  |
|  | β | SE | *P* |  |  | β | SE |  | β | SE |  | β | | SE | |  | | β | | SE | |  |
| rs4454603 | 0.23 | 0.07 | 4.25×10^-8^  1.52×10^-8^  6.35×10^-12^  4.90×10^-258^  1.63×10^-14^  1.14×10^-13^  7.39×10^-17^  7.49×10^-12^  6.02×10^-10^  1.46×10^-8^  1.23×10^-15^ | |  | 0.004 | 0.01 |  | -1×10^-4^ | 0.02 |  | -0.03 | 0.02 | |  | | 0.06 | | 0.02  0.03  0.02  0.02  0.02  0.03  0.05  0.06  0.03  0.03  0.02 | |  |  |
| rs10897169 | 0.27 | 0.08 |  |  |  | -0.01 | 0.01 |  | -0.01 | 0.03 |  | 0.004 | 0.02 | |  | | 0.004 | |  |  |  |  |
| rs13966 | 0.33 | 0.08 |  |  |  | -0.01 | 0.01 |  | 0.01 | 0.03 |  | -0.01 | 0.02 | |  | | 0.002 | |  |  |  |  |
| rs174541 | 1.45 | 0.06 |  |  |  | -0.02 | 0.01 |  | -0.10 | 0.03 |  | -0.02 | 0.02 | |  | | 0.03 | |  |  |  |  |
| rs1800009 | 0.34 | 0.07 |  |  |  | -0.01 | 0.01 |  | -0.01 | 0.03 |  | -0.001 | 0.02 | |  | | -0.02 | |  |  |  |  |
| rs198475 | 0.38 | 0.08 |  |  |  | -0.01 | 0.01 |  | -0.06 | 0.03 |  | 0.02 | 0.02 | |  | | -0.02 | |  |  |  |  |
| rs2521568 | 0.72 | 0.13 |  |  |  | -0.02 | 0.02 |  | -0.04 | 0.05 |  | -0.02 | 0.04 | |  | | 0.005 | |  |  |  |  |
| rs2521572 | 0.86 | 0.20 |  |  |  | -0.04 | 0.03 |  | -0.07 | 0.07 |  | -0.04 | 0.05 | |  | | 0.01 | |  |  |  |  |
| rs4963466 | 0.30 | 0.08 |  |  |  | 0.01 | 0.01 |  | 0.05 | 0.03 |  | 0.03 | 0.02 | |  | | 0.03 | |  |  |  |  |
| rs760306 | 0.28 | 0.08 |  |  |  | -0.02 | 0.01 |  | -0.005 | 0.03 |  | -0.04 | 0.02 | |  | | 0.03 | |  |  |  |  |
| rs16966952 | 0.35 | 0.07 |  |  |  | -0.001 | 0.01 |  | 0.03 | 0.03 |  | -0.03 | 0.02 | |  | | -0.03 | |  |  |  |  |
| CES, cardioembolic stroke; IS, ischemic stroke; LA, linoleic acid; LAS, large artery stroke; SE, standard error; SVS, small vessel stroke | | | | | | | | | | | | | | | | | | | | | | |

| **Supplementary Table 4** The reported associations between the instrumental SNPs and secondary traits from GWAS catalog search | |
| --- | --- |
| SNP | Reported traits |
| rs4454603 | waist-hip ratio |
| rs2521572 | plasma omega-3 polyunsaturated fatty acid levels |
| rs174541 | insulin sensitivity measurement, chronic kidney disease, serum metabolite measurement, fatty acid measurement, trans fatty acid measurement, arachidonic acid measurement, oleic acid measurement, docosapentaenoic acid measurement |

| **Supplementary Table 5** Associations of the three most significant SNPs with LA and IS with its subtypes | | | | | | | | | | | | | | | | | | | | | | | | | | | |
| --- | --- | --- | --- | --- | --- | --- | --- | --- | --- | --- | --- | --- | --- | --- | --- | --- | --- | --- | --- | --- | --- | --- | --- | --- | --- | --- | --- |
| SNP | Position | Chr | EA | Association with LA | | | |  | | Association with IS | | |  | | Association with LAS | | |  | | Association with CES | | |  | | Association with SVS | | |
|  |  |  |  | β | SE | *P* |  | | β | | SE |  | | β | | SE |  | | β | | SE |  | | β | | SE |  |
| rs10740118 | 65101207 | 10 | g | 0.25 | 0.04 | 8.08×10^-9^ |  | | 0.003 | | 0.01 |  | | -0.01 | | 0.02 |  | | -0.03 | | 0.02 |  | | 0.07 | | 0.02 |  |
| rs174547 | 61570783 | 11 | c | 1.47 | 0.04 | 4.98×10^-274^ |  | | -0.02 | | 0.01 |  | | -0.10 | | 0.03 |  | | -0.03 | | 0.02 |  | | 0.03 | | 0.03 |  |
| rs16966952 | 15135943 | 16 | a | 0.35 | 0.04 | 1.23×10^-15^ |  | | -0.001 | | 0.01 |  | | 0.03 | | 0.03 |  | | -0.03 | | 0.02 |  | | -0.03 | | 0.02 |  |
| CES, cardioembolic stroke; EA, effect allele; IS, ischemic stroke; LA, linoleic acid; LAS, large artery stroke; NEA non-effect allele; OR, odds ratio; SE, standard error; SVS, small vessel stroke. | | | | | | | | | | | | | | | | | | | | | | | | | | | |

| **Supplementary Table 6** Bidirectional Mendelian randomization associations between LA and IS with its subtypes based on the inverse variance-weighted method | | | | | | | |
| --- | --- | --- | --- | --- | --- | --- | --- |
| Exposure | Source for exposure^1^ | Outcome | Source for outcome^2^ | No. of SNPs | OR | 95% CI | *P* value |
| Ischemic stroke | MEGASTROKE consortium | linoleic acid | Kettunen et al. | 32 | 1.02 | (0.91,1.13) | 0.78 |
| Large artery stroke |  |  |  |  | 1.02 | (0.95,1.10) | 0.52 |
| Cardioembolic stroke |  |  |  |  | 1.02 | (0.97,1.08) | 0.46 |
| Small vessel stroke |  |  |  |  | 0.99 | (0.90,1.08) | 0.76 |
| CI: confidence interval; GWAS: genome-wide association study; OR: odds ratio.  ^1^Data sources and sample sizes: Genetic associations with ischemic stroke and its subtypes were derived from a GWAS of European ancestry (34,217 cases with  ischemic stroke and 404,630 controls) based on MEGASTROKE consortium.  ^2^ Summary statistics of linoleic acid were derived from a GWAS of European-descent individuals (n = 24,925). Reference: Kettunen J, Demirkan A, Wurtz P,  Draisma HH, Haller T, Rawal R, Vaarhorst A, Kangas AJ, Lyytikainen LP, Pirinen M, et al. Genome-wide study for circulating metabolites identifies 62 loci  and reveals novel systemic effects of LPA. Nature communications 2016;7:11122. doi: 10.1038/ncomms11122. | | | | | | | |

| **Supplementary Table 7** Associations between genetically predicted low density lipoprotein (LDL) cholesterol and ischemic stroke and its subtypes. | | | | |
| --- | --- | --- | --- | --- |
| Outcomes | Number of SNPs^a^ | OR (95% CI) | *P* value |  |
| IS | 209 | 1.08 (1.02-1.15) | 0.007 |  |
| LAS | 210 | 1.30 (1.15-1.47) | 2.68×10^-5^ |  |
| CES | 209 | 1.06 (0.97-1.16) | 0.198 |  |
| SVS | 209 | 1.03 (0.92-1.15) | 0.622 |  |
| *CES, cardioembolic stroke; CI, confidence interval; IS, ischemic stroke; IVW, inverse-variance weighted; LA, linoleic acid; LAS, large artery stroke; OR, odds ratio; SVS, small vessel stroke.*  *^a^ The instrumental SNPs for LDL cholesterol were obtained from the GWAS*  *multi-ethnic meta-analysis of the MVP and the GLGC samples (Klarin D et al., Nat Genet 2018;50:1514-23).* | | | | |

| **Supplementary Table 8** Multivariable MR analysis of the association between predicted linoleic acid (LA) and ischemic stroke and its subtypes by adjusting for low density lipoprotein (LDL) cholesterol. | | | | |
| --- | --- | --- | --- | --- |
| Outcomes | Number of SNPs^a^ | OR (95% CI) | *P* value |  |
| IS | 11 | 0.99 (0.96-1.01) | 0.130 |  |
| LAS | 11 | 0.95 (0.90-1.01) | 0.105 |  |
| CES | 11 | 1.00 (0.96-1.04) | 0.905 |  |
| SVS | 11 | 1.04 (0.99-1.09) | 0.156 |  |
| *CES, cardioembolic stroke; CI, confidence interval; IS, ischemic stroke; IVW, inverse-variance weighted; LA, linoleic acid; LAS, large artery stroke; OR, odds ratio; SVS, small vessel stroke.* | | | | |

**
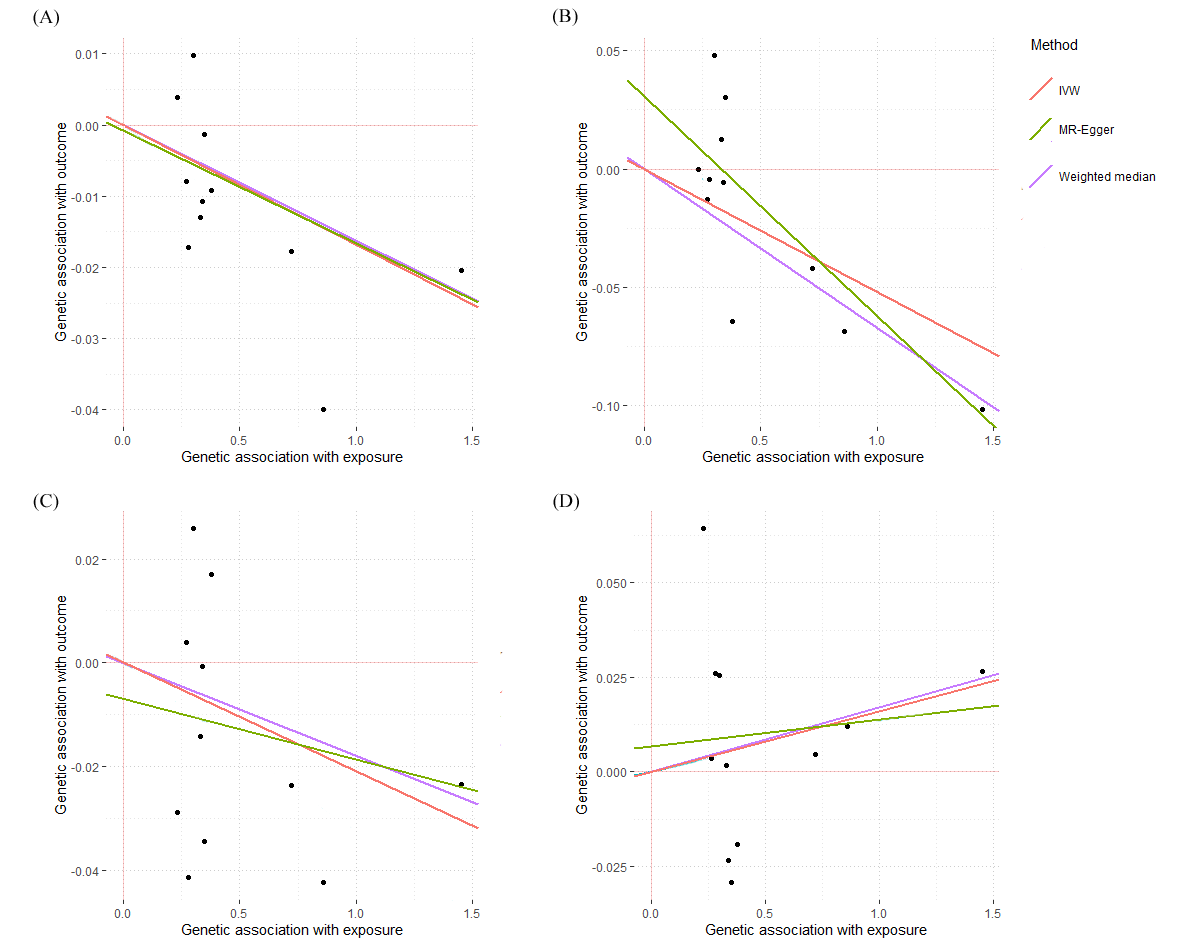
**

**Supplementary Figure 1** Scatter plot of the causal effects of LA associated single-nucleotide polymorphisms on the risk of (a) ischemic stroke, (b) large-artery stroke, (c) cardioembolic stroke, and (d) small vessel stroke. Red line indicates MR analysis by IVW method, and blue line and purple line are used to present MR results of MR-egger regression and weighted median method, respectively.

**Members of MEGASTROKE consortium**

We gratefully acknowledge the contributing studies and databases (MEGASTROKE project) that made GWAS summary data available. We acknowledge the contributions of Malik R, Chauhan G, Traylor M, Sargurupremraj M, Okada Y, Mishra A, Rutten-Jacobs L, Giese AK, van der Laan SW, Gretarsdottir S, Anderson CD, Chong M, Adams HHH, Ago T, Almgren P, Amouyel P, Ay H, Bartz TM, Benavente OR, Bevan S, Boncoraglio GB, Brown RD Jr, Butterworth AS, Carrera C, Carty CL, Chasman DI, Chen WM, Cole JW, Correa A, Cotlarciuc I, Cruchaga C, Danesh J, de Bakker PIW, DeStefano AL, den Hoed M, Duan Q, Engelter ST, Falcone GJ, Gottesman RF, Grewal RP, Gudnason V, Gustafsson S, Haessler J, Harris TB, Hassan A, Havulinna AS, Heckbert SR, Holliday EG, Howard G, Hsu FC, Hyacinth HI, Ikram MA, Ingelsson E, Irvin MR, Jian X, Jiménez-Conde J, Johnson JA, Jukema JW, Kanai M, Keene KL, Kissela BM, Kleindorfer DO, Kooperberg C, Kubo M, Lange LA, Langefeld CD, Langenberg C, Launer LJ, Lee JM, Lemmens R, Leys D, Lewis CM, Lin WY, Lindgren AG, Lorentzen E, Magnusson PK, Maguire J, Manichaikul A, McArdle PF, Meschia JF, Mitchell BD, Mosley TH, Nalls MA, Ninomiya T, O'Donnell MJ, Psaty BM, Pulit SL, Rannikmäe K, Reiner AP, Rexrode KM, Rice K, Rich SS, Ridker PM, Rost NS, Rothwell PM, Rotter JI, Rundek T, Sacco RL, Sakaue S, Sale MM, Salomaa V, Sapkota BR, Schmidt R, Schmidt CO, Schminke U, Sharma P, Slowik A, Sudlow CLM, Tanislav C, Tatlisumak T, Taylor KD, Thijs VNS, Thorleifsson G, Thorsteinsdottir U, Tiedt S, Trompet S, Tzourio C, van Duijn CM, Walters M, Wareham NJ, Wassertheil-Smoller S, Wilson JG, Wiggins KL, Yang Q, Yusuf S, Amin N, Aparicio HS, Arnett DK, Attia J, Beiser AS, Berr C, Buring JE, Bustamante M, Caso V, Cheng YC, Choi SH, Chowhan A, Cullell N, Dartigues JF, Delavaran H, Delgado P, Dörr M, Engström G, Ford I, Gurpreet WS, Hamsten A, Heitsch L, Hozawa A, Ibanez L, Ilinca A, Ingelsson M, Iwasaki M, Jackson RD, Jood K, Jousilahti P, Kaffashian S, Kalra L, Kamouchi M, Kitazono T, Kjartansson O, Kloss M, Koudstaal PJ, Krupinski J, Labovitz DL, Laurie CC, Levi CR, Li L, Lind L, Lindgren CM, Lioutas V, Liu YM, Lopez OL, Makoto H, Martinez-Majander N, Matsuda K, Minegishi N, Montaner J, Morris AP, Muiño E, Müller-Nurasyid M, Norrving B, Ogishima S, Parati EA, Peddareddygari LR, Pedersen NL, Pera J, Perola M, Pezzini A, Pileggi S, Rabionet R, Riba-Llena I, Ribasés M, Romero JR, Roquer J, Rudd AG, Sarin AP, Sarju R, Sarnowski C, Sasaki M, Satizabal CL, Satoh M, Sattar N, Sawada N, Sibolt G, Sigurdsson Á, Smith A, Sobue K, Soriano-Tárraga C, Stanne T, Stine OC, Stott DJ, Strauch K, Takai T, Tanaka H, Tanno K, Teumer A, Tomppo L, Torres-Aguila NP, Touze E, Tsugane S, Uitterlinden AG, Valdimarsson EM, van der Lee SJ, Völzke H, Wakai K, Weir D, Williams SR, Wolfe CDA, Wong Q, Xu H, Yamaji T, Sanghera DK, Melander O, Jern C, Strbian D, Fernandez-Cadenas I, Longstreth WT Jr, Rolfs A, Hata J, Woo D, Rosand J, Pare G, Hopewell JC, Saleheen D, Stefansson K, Worrall BB, Kittner SJ, Seshadri S, Fornage M, Markus HS, Howson JMM, Kamatani Y, Debette S, Dichgans M.
